# Community case management of malaria using ACT and RDT in two districts in Zambia: achieving high adherence to test results using community health workers

**DOI:** 10.1186/1475-2875-10-158

**Published:** 2011-06-09

**Authors:** Pascalina Chanda, Busiku Hamainza, Hawela B Moonga, Victor Chalwe, Franco Pagnoni

**Affiliations:** 1 Department of Public Health and Research, Ministry of Health Headquarters, Lusaka, Zambia; 2 Operational Research Unit, National Malaria Control Centre, Lusaka, Zambia; 3 Parasitology Unit, National Malaria Control Centre, Lusaka, Zambia; 4 Department of Clinical Sciences, Tropical Diseases Research Centre, Ndola, Zambia; 5 UNICEF/UNDP/World Bank/WHO Special Programme for Research and Training in Tropical Diseases, World Health Organization, Geneva, Switzerland

## Abstract

**Background:**

Access to prompt and effective treatment is a cornerstone of the current malaria control strategy. Delays in starting appropriate treatment is a major contributor to malaria mortality. WHO recommends home management of malaria using artemisininbased combination therapy (ACT) and Rapid Diagnostic tests (RDTs) as one of the strategies for improving access to prompt and efective malaria case management.

**Methods:**

A prospective evaluation of the effectiveness of using community health workers **(**CHWs) as delivery points for ACT and RDTs in the home management of malaria in two districts in Zambia.

**Results:**

CHWs were able to manage malaria fevers by correctly interpreting RDT results and appropriately prescribing antimalarials. All severe malaria cases and febrile non-malaria fevers were referred to a health facility for further management. There were variations in malaria prevalence between the two districts and among the villages in each district. 100% and 99.4% of the patients with a negative RDT result were not prescribed an antimalarial in the two districts respectively. No cases progressed to severe malaria and no deaths were recorded during the study period. Community perceptions were positive.

**Conclusion:**

CHWs are effective delivery points for prompt and effective malaria case management at community level. Adherence to test results is the best ever reported in Zambia. Further areas of implementation research are discussed.

## Background

Access to prompt and effective treatment is a cornerstone of the current malaria control strategy [1]. Delays in starting appropriate treatment is a major contributor to malaria mortality. Many children with suspected malaria in sub-Saharan Africa, where medical services are not easily accessible, start treatment too late or do not receive it at all and die at home without contact with formal medical services [2,3]. It is for this reason that the World Health Organization has recommended home management of malaria (HMM) as one of the strategies for improving access to prompt and effective malaria case management [4]. Further, community referral compliance rates observed in some studies indicate the possibility detecting malaria episodes early, initiating pre-health facility management of cases and improving outcomes of severe malaria cases at health facility [5].

Home management of malaria with chloroquine has been effective in reducing both mortality and severe malaria morbidity [6,7]. However, due to parasite resistance to monotherapies, the use artemisinin-based combination therapy (ACT) in HMM is being promoted and has shown great potential of being used safely in the community. The feasibility and acceptability of the use of ACT within the context of HMM has been demonstrated in Ghana [8] and in a multi-centre study involving four African sites [9]. Furthermore, scaling-up of the HMM strategy has been shown to reduce workload in health facilities [10] and might thus contribute to minimize the consequences of workforce shortage at peripheral health facilities level, and possibly improve their performance

In Zambia, the first line malaria treatment policy was changed from chloroquine to the combination artemether-lumefantrine (AL) due to parasite resistance to monotherapies [11]. However, at community level, community health workers (CHWs) are still using sulphadoxine-pyrimethamine (SP) for treating uncomplicated malaria. SP has showed decreasing efficacy rates with treatment failures estimated to be 30% [12]. CHWs play a big role in the provision of very basic health services in areas with geographical access challenges and where there is a shortage of professional health workers in rural areas [13,14]. Within the context of the health system in Zambia, the community level is defined as the level that *'brings health care as close to the family as possible' *(National Health Strategic Plan 2006-2010). In malaria control, CHWs are expected to promote ITNs and other preventive measures such as environmental management, provide individual counselling and group education, detect cases of fever and pregnancy, provide simple case management and prophylaxis, and know when to refer in all cases and circumstances [15].

Having successfully reached countrywide coverage up to facility level, plans are underway to incorporate the community level as a service delivery point for malaria case management using ACT and RDTs on a national scale. It is against this background that this pilot study was conducted to generate information on the capacity of CHWs to use RDTs and ACT as effective tools for HMM. It was anticipated that results from this study could inform policy on the feasibility and effectiveness of a large scale HMM programme implemented using CHWs. An important component of the above objective was to assess whether this model would achieve good anti-malarial prescription practices in relation to the diagnostic test results for malaria.

## Methods

This was a prospective evaluation of the home management of malaria by CHWs using RDT and ACT. The study was conducted in two districts, which had baseline data on the home management of malaria practices and acceptability. Chongwe district is located in Lusaka Province of Zambia. The area is typically rural and experiences moderate malaria transmission. The district is estimated to have a population of about 157,664 inhabitants. There are 26 health facilities in the district, annual malaria incidence was estimated at 130/1000 population in 2008 (HMIS 2007). Kalomo district on the other hand is situated in the Southern Province of Zambia. The district has a population of about 181,379 people. The district has 24 health facilities and the annual malaria incidence was estimated to at 82/1000 population [16]. Both districts are implementing the user fees removal policy (thus patients are not expected to pay any fees for the basic health care package).

Originally the study was designed for children under five years, however, all age groups sought care through the CHWs models in line with the national health care system. CHWs act as first level contact in hard to reach areas (accessibility). Therefore, the project captured all age groups attended to by the CHWs. The sample included all cases reporting to the CHWs in the study sites between June 2008 and December 2009. The study period covered both high and low transmission seasons.

Initially, the CHWs and health facility staff who were in the study sites received standard training on malaria case management based on the national guidelines on the new malaria treatment policy. The trainings were tailored for CHWs and health facility staff respectively. The trainings included practical sessions on how to perform malaria testing using RDTs and how to interpret the results. Additional training included stock management and infection prevention. Emphasis was placed on using the diagnostic result to inform the decision whether to prescribe an anti-malarial or not for uncomplicated malaria, referral mechanisms for severe malaria cases and non-malaria febrile cases.

Malaria registers were developed for use by CHWs to collect morbidity and mortality data. This was a basis for records review regarding completeness, workload, diagnostic results and number of cases managed or referred. The CHWs were oriented on how to fill the registers to ensure completeness of records.

### Diagnostic testing

For this study, the HRP-2 antigen based RDT, specific for *Plasmodium falciparum *was used at all study sites in line with the national stocks. The parasitological profile data collected by the malaria programme have shown no occurrence of other forms of malaria, either single or as mixed infections, in both Chongwe and Kalomo districts (NMCC 2008 unpublished). The cases were managed based on the CHW algorithm for fever (NMCC 2004 unpublished) and the RDT job aids. Training on RDT use was conducted prior to the study. Quality assurance mechanisms were put in place, both retrospectively and prospectively, to ensure the RDT tests were functioning properly but also to ensure the tests were being performed correctly.

### Pre-packed anti-malarial

The current malaria drug policy in Zambia is the use of a six-dose AL, as a combination treatment for uncomplicated malaria. The drug is co-formulated and co-packaged. CHWs in Zambia have been trained on the new malaria treatment policy. Further training was provided prior to the commencement of the study on how to prescribe AL.

### Record reviews

Record reviews were conducted based on the documentation available in the registers provided to CHWs and health facility staff. A health facility and CHWs checklist was used to collect data on fever/malaria cases, confirmatory test result, treatment options and outcome of treatment including referrals.

### Focus group discussion and in-depth interviews

In all about 10 focus group discussions (FGDs) were conducted in the study areas. Although extensive field notes were taken during interviews, all interviews were recorded and transcribed. FGDs were conducted among care-takers, health facility personnel and CHWs. In depth interviews were also conducted with care-takers and CHWs.

### Field observations

The field observations were performed to address the feasibility of CHWs to correctly use the job aids for RDTs, which were developed to assist with use and interpretation of results. This was to provide insights for improving upon future versions of job aids and also contribute to assessing the applicability of written RDT instructions for CHWs use. Other objectives of field observations were to identify the capacity of CHWs to correctly administer ACT to patients in whom malaria was diagnosed, and their ability to identify signs and symptoms of uncomplicated and severe malaria.

### Data analysis

The quantitative surveys data was entered in excel and analysed using STATA version 8. For the qualitative study, data was analysed by themes and sub-themes using the grounded theory approach. Data was organized to detect themes, codes and contents.

### Ethical consideration

Ethical approval was obtained from the Tropical Disease Research Ethics Committee. Further clearance was obtained from the Permanent Secretary of the Ministry of Health.

### Consent

Written informed consent was obtained from the study participants. A copy of the written consent is available for review by the Editor-in-Chief of this journal.

## Results

### Number and proportion of patients visiting CHWs

Patient visits at the two study sites showed that between June 2008 and December 2009, a total of 9,847 fever cases were seen by CHWs on the study. The number of fever cases seen by CHWs was 5648 in Chongwe and 4199 in Kalomo. Not all the fever cases however were found to have malaria, as shown in Table 1. There were more female patients than were males in both districts. This is consistent with the ZDHS 2007 estimates which show that the Zambian population has more females than males (52% vs 48%) respectively [17].

**Table 1:** Characteristics and outcomes of patients visiting CHWs

Characteristics	Chongwe N (%)	Binomial Exact 95% CI	Kalomo N (%)	Binomial Exact 95% CI
**No of CHWs evaluated**	9		7	

**Catchment population for CHWs**	16,079		18,279	

**Number of patient visits**	5648 (35.1%)	[34.4-35.9]	4199 (23.0%)	[22.4-23.6]

**Sex:**				

**Male**	2547 (45.1%)	[43.8-46.4]	1864 (44.4%)	[42.9-45.9]

**Female**	3101 (54.9%)	[53.6-56.2]	2335 (55.6%)	[54.1-57.1]

**Number with positive RDT (with or without signs of severe malaria)**	257 (4.5%)	[4.0-5.1]	2061 (49.1%)	[47.6-50.6]

**Number with signs of severe malaria at first contact**	12 (4.7%)	[2.4-8.0]	8 (0.4%)	[0.2-0.8]

**Uncomplicated malaria cases treated with AL**	245 (100%)	[98.5-100]	2037 (99.2%)	[98.7-99.6]

**Uncomplicated malaria cases treated with SP**	0 (0%)	-	16 (0.8%)	[0.4-1.2]

**Cases with negative RDT treated with antimalarial**	0 (0%)	-	13 (0.6%)	[0.3-1.1]

**Number of malaria deaths at CHW**	0		0	

**Number of referrals to health facility for further management**	80		735	

**Number confirmed to have reached referral**	34 (42%)	[31.5-54.1]	294 (40%)	[36.4-43.6]

In Chongwe district, the proportion of fever cases found to have malaria was 4.5% (257/5648) while in Kalomo malaria prevalence was very high at 49.1% (2061/4199). The diagnostic tool used in both district was an HRP-2 based RDT. HRP-2 based RDTs, when used in high parasite prevalence regions, have a tendency of overestimating the disease burden by approximately 10% [18]. This phenomenon is an important aspect of the test because it reduces the probability of missing a true positive malaria case and hence does not pose a danger if a negative case for malaria is not treated with antimalarial. However, this may delay investigation and alternative treatment in non-malaria febrile cases that are erroneously considered as malaria. In Chongwe all the patients were subjected to an RDT test, while in Kalomo 3% were clinically diagnosed to have malaria, due to a short period of RDT stock-out.

The malaria prevalence in Kalomo was found be high compared to Chongwe and also well above the national average for 2008 and 2010 malaria indicator surveys (10% and 16%) respectively. The malaria prevalence across villages in the same district was found to be varied as shown in Table 2.

**Table 2:** Parasite rates (%) by CHW

	Chongwe	Kalomo
**CHW1**	17.5	55.1

**CHW2**	3.5	55.8

**CHW3**	0.5	59.7

**CHW4**	0.4	74.4

**CHW5**	1.0	31.6

**CHW6**	2.3	4.2

**CHW7**	3.6	31.1

**CHW8**	1.3	

**CHW9**	2.6	

From Table 1, 35% of the catchment population was seen by CHWs in Chongwe district while only 23% sought care through the CHWs in Kalomo. In both districts, the villages served by CHWs are not very close to the health facility. All CHWs in Kalomo were males while in Chongwe, two out of nine were female. All CHWs who were in this study were found to have attained up to secondary level education and they were all above 35 years old.

### Antimalarial prescription patterns

The CHWs were trained only to prescribe anti-malarials to confirmed malaria cases and refer other non-malaria patients to the nearest health facility for further management. In order to do this, CHWs were required to screen the patients with fever or history of fever using their algorithm for patient management. When the RDTs was negative and other casue of illness was suspected, the patient was then referred to the nearest heath facility so that appropriate tests and treatment could be provided.

For cases not needing referral, according to the CHWs assessments, the most common prescriptions were pain killers, multivitamins or de worming tablets. In Chongwe, all malaria patients (positive RDT result) were treated with AL. No anti-malarial was prescribed to cases with negative RDT. In Kalomo, among the positive cases, 99.2% received the drug of choice AL while 0.8% were treated with SP. A few cases (0.6%) with negative RDT were treated with AL. The practice observed here is consistent with the guidelines on good case management and shows that CHWs, if well trained and monitored, coupled with the use of job aids, are able to correctly use and interpret RDTs and appropriately prescribe ACT.

All the CHWs reported no stockouts of the first-line treatment. However, in Kalomo district, there was stock out of RDTs for less than two weeks in May 2009.

During the field observations and by reviewing records and matching the anti-malarial dosage with the patient age, it was found that all CHWs were able to prescribe the pre packaged anti-malarial correctly. This is also corroborated by the findings above where anti-malarials were nearly exclusively prescribed to those who were found to be carrying malaria parasites as opposed to any fever case.

This adherence to the test result is the best ever malaria case management practice reported in Zambia even among professional health workers. The non-adherence to RDT results is a major issue in malaria case management in the country [19,20]. The training and supervision model used in this programme coupled with quality assurance systems and continuous availability of the testing tool has the potential to improve adherence to diagnostic test results and overall malaria case management. This has spill over effects to good patient management in general through provision of the appropriate alternative treatment either by CHW or at health facility.

### Proportion of cases seen by CHWs progressing to severe malaria

Malaria, if diagnosed early and promptly treated with an effective drug can neither progress to severe malaria nor lead to death. In this study, all the 20 severe malaria cases were referred to the health facility. And to assess progress of the patients, all reviews were conducted within 3 days of commencing treatment to ensure that no false negatives were left untreated. However, in this study, no false negatives were found. This is consistent with findings at facility level reported by Chanda *et al *in 2009 [21].

At the end of the study no severe malaria cases were reported in patients initially diagnosed as uncomplicated malaria at all the sites. The only severe malaria cases reported were those identified at the screening process (12 in Chongwe and 8 in Kalomo) and these were referred to the health facility for treatment with quinine as per national treatment guidelines. The differences in the rate of severe malaria reported in the districts may be due to differences in prevalence.

### Ability of the CHWs to use the referral mechanisms

The CHWs catchment area is linked to the health facility under which they fall. The health facility provides logistics and supplies for the routine work of the CHWs. In the project model, CHWs were obtaining drugs and RDTs through the health facilities. The health facilities were trained on supervision and feedback mechanisms for referrals from CHWs.

Of the 735 referrals sent for further management to the health facility in Kalomo district, CHWs received feedback for 40% of the patients. In Chongwe district, feedback was provided to CHWs for 42% of the 80 patients referred. However, this is the proportion confirmed to have actually reached the health facility by health centre staff through a written note. During the FGDs, CHWs reported that health workers do not always provide written feedback for referrals sent to them. This leads to work overload for CHWs who have to do home visits to ensure that all the referred patients went to their place of referral.

The referral system could be more effective if the two-way communication system is maintained by both CHWs and health workers.

### Capacity of health workers to supervise CHWs drug and logistics

The logistics system for CHWs for this project was based on an existing system where limited supplies for two weeks were issued by the health centre staff. A simple logistics form of issues and receipt was maintained and that was used by both parties when new requests were made.

During the study, it was found that health workers were able to ensure that no stock outs occurred. The only stock outs which occurred for RDTs in Kalomo district (less than two weeks) was due to a shortage in the whole district and not at health centre level. The health centre staff were trained through the project on drug and logistics management including supervisions of CHWs.

In Chongwe, all but one CHW reported receiving monthly supervisory visits. In Kalomo, the supervisory visits by health workers were reported to take have taken place once every quarter (four times in a year). In Chongwe district, three CHWs were invited to work at the health facility under the supervision of the health centre staff to ensure that they were complying with the patient management requirements. This approach however was not liked by CHWs, as they felt that they were manning health centres at the expense of them providing care in their home villages.

## Discussion

Access to effective and timely diagnosis and treatment for malaria within 24 hours of symptom onset is a key intervention in malaria control efforts. It must, however, be noted that the low specificity of presumptive diagnosis has implications for the deployment of ACT at the community level hence the need for deployment of parasite based diagnostic tools in tandem with ACT deployment [22]. The study endeavoured to demonstrate the viability of using CHWs as service delivery points for malaria case management using HRP-2 based RDTs and ACT. Over the 18 months of the study, CHWs attended to patients of all ages and sexes indicating that communities use these individuals for health care delivery. In Chongwe CHWs attended to an estimated 35% of the catchment population, while Kalomo had 23%. The majority of the patients were children under the age of five. As these are patients that might have sought care from the health facilities, our study confirms the potential of the HMM strategy to reduce the case load of patients at the health facilities, as previously indicated by other studies [23,10].

The CHWs were found to adhere to established guidelines for malaria diagnosis and treatment. From the study only 0.6% of the patients received treatment for a negative RDT result. However all the CHWs prescribed the dosages correctly. This is consistent with other findings on the ability of CHWs to manage uncomplicated malaria within their communities [24]. The demonstrated adherence to the test results in this study is the best ever malaria case management practice reported in Zambia even among professional health workers. The non-adherence to RDT results is major issue in malaria case management in the country [19,20]. The training and supervision model used in this programme coupled with quality assurance systems and continuous availability of the testing tool has the potential to improve adherence to diagnostic test results and overall malaria case management. This has spill over effects to good patient management in general. A challenge that was observed from this study is that of patient demands, where CHWs reported that some patients still demanded anti-malarial treatment in spite of a negative RDT result. Thus, information on confirmatory malaria diagnosis should not be limited to health personnel or workers only but should be extended to the general population.

During the study no deaths were reported. This corroborates the finding that all the severe malaria cases which were identified by CHWs at the screening process were referred to the health facility as per training guidelines. The relatively low caretaker compliance rate with the referral advice is a matter of concern. However, the rate of 40-42% may be underestimated due to the source of information (written feedback). During FGDs, the CHWs reported that not all health workers provided written feedback, thereby making it difficult for the CHWs to correctly estimate the number of referrals who complied with the advice given to them. During FGDs, it was found that some care-takers did not like to be referred to the health facilities because of long waiting hours and long distances in some cases. The CHWs also commented on the fact that health centre staff were not consistent in providing the CHWs with specific patient feedback after receiving referrals. This shifts the workload to CHWs who have to do home visits to ensure that all the referred patients went to their place of referral. The referral system could be more effective if the two-way communication system is maintained by both CHWs and health workers and more patient education is done on the importance of complying with the referral advice.

There are variations with regard to parasite rates detected by CHWs and during the different months of the year. The parasite rates variations may be due to variations in locations of the villages across each district. This has implications in overall malaria control as this shows that control efforts should be focal and take into account the factors that lead to malaria transmisiion in each of the villages in a given district.

The key health concerns expressed by the CHWs included malaria, HIV/AIDS, diarrhoea, coughs and eye infections. The study did not rank these from the respondents perspective, however for all excluding malaria, CHWs said they could not do much to help their communities because they did not have the required tools for treatment. It is, therefore, important to consider the idea of integrated community case management (iCCM), which aims to empower CHWs with skills and tools to manage more ailments at a commmunity level.

All the CHWs received training on use of RDTs and ACT. During the training they were taught the signs and symptoms of malaria, which on assessment a year later they could remember very well. This confirms that a lay healthworker trained in a systematic and simple manner can retain information about how a disease may manifest and be able to provide appropriate treatment. All the CHWs had job aids for ACT and RDTs, which they indicated they used occasionally during their work. The RDT job aids which were provided were translated into the major local languages. However, all CHWs said they were comfortable using the English version.

The CHWs also showed ability to manage the stocks of commodities; no RDTs or ACT expired during the study period. A brief RDT stock out was experienced by the CHWs due to a national stockout of RDTs. Additionally, all the CHWs were able to track the use of their commodities and make orders for restocking in a timely manner.

The care givers exhibited a fair understanding of the symptoms of malaria and the prevention methods. In addition they were able to articulate the methods for fever management. Caregivers indicated that they had challenges with access to health facilities due to staff shortages, and that they were happy with the services provided by the CHWs. This is indicative of the need/demand for services offered by the CHWs, as close to the home as possible. Caregivers were asked about their perception of services rendered by CHWs. It was generally agreed that people were happy with the services, and the way they were being delivered by the CHWs. The attitude of CHWs was described as "perfect" because they were reported to be committed to attending to patients. The high care taker acceptability of CHWs may also be attributed to the fact that the community members are the ones who recommend a given individual to be trained by health centre personnel to work as a CHW.

The CHW initiative is cardinal to improving health service delivery systems [13,14]. The study has shown that this strategy is feasible, acceptable by communities and effective for malaria case management. In order for the intitaive to work, there is need to ensure the availability of commodities required by the CHWs to test and treat malaria at all times. Furthermore, even though the CHWs have been working as volunteers, there is need to provide sustainable incentives for their work due to the fact that the number of patients they see increases when they are able to offer more health services. Additionally, there were some patients that had fever but did not have malaria and these where refered for further management to the health facility. In order to provide more comprehensive health services the concept of community case management needs to be further developed, CHWs to be able to diagnose and treat various uncomplicated ailments such as diarrhoea and pneumonia especially in children. There is also need to develop mechanisms to improve the refferral system between the CHW and health facility.

Further implementation research is required to develop effective referral mechanisms in HMM. There is also alot more work required to understand the impact of HMM startegy on non-malaria fever outcomes and also to understand how to improve the caretaker adherence to referral advice.

## Conclusion

The study showed that community case management of malaria by CHWs using RDTs and ACT is feasible, acceptable by the communities and efficient. CHWs were able to manage appropriately malaria related fever episodes, including referral of cases for further management at the health facility. The CHWs adhered to the RDT result and only prescribed anti-malarials to cases with parasitological confirmation. The adherence to RDT test result found in this study was found to be the best practice reported in the country so far. This model has thus the potential to improve malaria case management and lead to cost savings by reducing over prescription of anti-malarials. It has also the potential to improve overall disease management, since it provides an opportunity for non-malaria patients to be investigated for other causes of fever. However, referral mechanisms need strengthening to ensure that non-malaria fevers get the appropriate treatment they need timely.

Scaling up malaria case management with ACT and RDTs coupled with intensified training and supervision has the potential to improve malaria case management in the remote parts of Zambia.

## Competing interests

The authors declare that they have no competing interests.

## Authors' contributions

PC was responsible for the proposal development, study administration, data analysis and manuscript development. BH, MH and VC participated equally in proposal development, data collection, data entry and analysis and manuscript development. FP provided invaluable input at proposal development, data analysis and manuscript development. All authors read and approved the final manuscript.

## References

[B1] World Health Organization/UNICEF Africa Malaria Report 2003. WHO, Geneva 2003 http://mosquito.who.int/amd2003.

[B2] Velema JP Alihonou EM Gandaho T Hounye FH Childhood mortality among users and non-users of primary health care in a rural west African community Int J Epidemiol 1991 20 474 479 10.1093/ije/20.2.474 1917252

[B3] Ministry of Health Tanzania The Adult Morbidity and Mortality Project Dar es Salaam, MoH, AMMP project 2004

[B4] World Health Organization Scaling up home-based management of malaria: from research to implementation 2004 Geneva: Switzerland

[B5] Källander K Tomson G Nsungwa-Sabiiti J Senyonjo Y Pariyo G Stefan Peterson S Community referral in home management of malaria in western Uganda: A case series study BMC Int Health Hum Rights 2006 6 2 10.1186/1472-698X-6-2 16539744 PMC1434779

[B6] Kidane G Morrow RH Teaching mothers to provide home management of malaria in Tigray, Ethopia: a randomized trial Lancet 2000 356 550 555 10.1016/S0140-6736(00)02580-0 10950232

[B7] Sirima SB Konaté A Tiono AB Convelbo Cousens S Pagnoni F Early treatment of childhood fevers with pre-packed antimalarial drugs in the home reduces severe malaria morbidity in Burkina Faso Trop Med Int Health 2003 8 133 139 10.1046/j.1365-3156.2003.00997.x 12581438

[B8] Chinbuah AM Gyapong JO Pagnoni F Wellington EK Gyapong M Feasibility and acceptability of the use of artemether-lumefantrine in the home management of uncomplicated malaria in children 6-59 months old in Ghana Trop Med Int Health 2006 11 1003 1016 10.1111/j.1365-3156.2006.01654.x 16827701

[B9] Ajayi IO Browne EN Garshong B Bateganya F Yusuf B Agyei-Baffour P Doamekpor L Balyeku A Munguti K Cousens S Pagnoni F Feasibility and acceptability of artemisinin-based combination therapy for the home management of malaria in four African sites Malar J 2008 7 6 10.1186/1475-2875-7-6 18182114 PMC2254635

[B10] Tiono AB Kaboré Y Traoré A Convelbo N Pagnoni F Sirima SB Implementation of Home based management of malaria in children reduces the work load for peripheral health facilities in a rural district of Burkina Faso Malar J 2008 7 201 10.1186/1475-2875-7-201 18834504 PMC2570683

[B11] Chanda P Masiye F Chitah BM Naawa S Moonga HB Banda P Okorosobo T A cost-effectiveness analysis of artemether lumefantrine for treatment of uncomplicated malaria in Zambia Malar J 2007 6 21 10.1186/1475-2875-6-21 17313682 PMC1804270

[B12] Chanda P Sikaala CH Kapelwa W Moonga H Njunju E Macdonald M Thea D Hamer DH Sipilanyambe N Assessment of the therapeutic efficacy of artemether-lumefantrine (Coartem®) and sulphadoxine-pyrimethamine (SP)-artesunate in Zambian children. 53rd Annual Meeting of the Society of Tropical Medicine and Hygiene 2004 Miami, FL. Abstract 21319735557

[B13] Haines A Sanders D Lehmann U Rowe AK Lawn JE Jan S Walker DG Bhutta Z Achieving child survival goals: potential contribution of community health workers Lancet 2007 369 2121 31 10.1016/S0140-6736(07)60325-0 17586307

[B14] Rosato M Laverack G Grabman H Tripathy P Nair N Mwansambo C Aza K Morrison J Bhutta Z Perry H Rifkin S Costello A Community participation: lessons for maternal, newborn, and child health Lancet 2008 372 962 971 10.1016/S0140-6736(08)61406-3 18790319

[B15] National Malaria Control Centre Roll Back Malaria Strategic Plan 2001-2005 2001 Central Board of Health, Zambia

[B16] Ministry of Health Annual Health Statistics Bulletin, Health Management Information System 2007 2007 Lusaka, Zambia

[B17] ZDHS 2007

[B18] Keating J Miller JM Bennet A Moonga HB Eisele TP *Plasmodium falciparum *parasite infection prevalence from a household survey in Zambia using microscopy and a rapid diagnostic test: implications for monitoring and evaluation Acta Trop 2009 112 277 282 10.1016/j.actatropica.2009.08.011 19682968

[B19] Zurovac D Ndhlovu M Sipilanyambe N Chanda P Hamer DH Simon JL Snow RW Paediatric malaria case management with artemether-lumefantrine in Zambia: a repeat cross sectional study Malar J 6 31 10.1186/1475-2875-6-31 PMC1832199 17367518

[B20] Hamer DH Ndhlovu M Zurovac D Fox M Yeboah-Antwi K Chanda P Naawa S Simon JL Snow RW Improved Diagnostic Testing and Malaria Treatment Practices in Zambia JAMA 2007 297 2227 2231 10.1001/jama.297.20.2227 17519412 PMC2674546

[B21] Chanda P Hamainza B Mulenga S Chalwe V Msiska C Chizema-Kawesha E Early results of integrated malaria control and implications for the management of fever in under-five children at a peripheral health facility: a case study of Chongwe rural health centre in Zambia Malar J 2009 8 49 10.1186/1475-2875-8-49 19292919 PMC2662870

[B22] Ajayi IO Falade CO Kale OO An assessment of accuracy of mothers' presumptive diagnosis of fever at home in southwest Nigeria: evidence for switch to parasite-based diagnostic test East Afr J Public Health 2009 6 229 234 20803910

[B23] Lemma H Byass P Desta A Bosman A Costanzo G Toma L Fottrell E Marrast A Ambachew Y Getachew A Mulure N Morrone A Bianchi A Barnabas GA Deploying artemether-lumefantrine with rapid testing in Ethiopian communities: impact on malaria morbidity, mortality and healthcare resources Trop Med Int Health 2010 15 241 250 10.1111/j.1365-3156.2009.02447.x 19961564

[B24] Hopkins H Talisuna A Whitty JMC Staedke SG Impact of home-based management of malaria on health outcomes in Africa: a systematic review of the evidence Malar J 2007 6 134 10.1186/1475-2875-6-134 17922916 PMC2170444

